# Tuning the Encapsulation of Simple Fragrances with an Amphiphilic Graft Copolymer

**DOI:** 10.1021/acsami.0c05892

**Published:** 2020-05-28

**Authors:** Marianna Mamusa, Constantina Sofroniou, Claudio Resta, Sergio Murgia, Emiliano Fratini, Johan Smets, Piero Baglioni

**Affiliations:** † Department of Chemistry “Ugo Schiff” and CSGI, University of Florence, Via della Lastruccia 3, 50019 Sesto Fiorentino, Italy; ‡ Dipartimento di Scienze Chimiche e Geologiche, Università degli Studi di Cagliari, S.S. 554 Bivio per Sestu, 09042 Monserrato, Italy; § The Procter & Gamble Company, Temselaan 100, 1853 Strombeek-Bever, Belgium

**Keywords:** encapsulation, amphiphilic polymer, fragrance, phase diagram, small-angle X-ray scattering

## Abstract

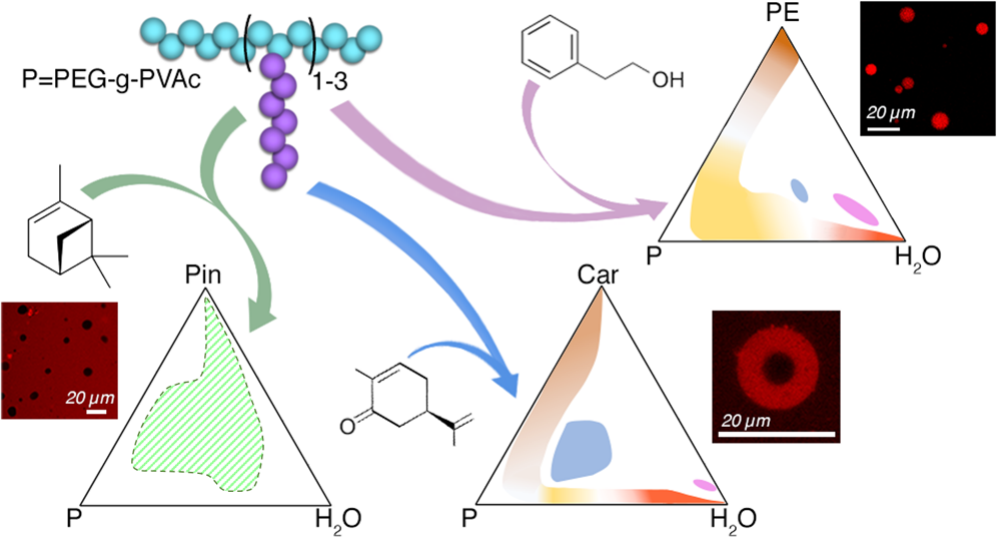

The encapsulation of poorly water-soluble compounds such as perfumes, flavors, and bioactive molecules is a key step in the formulation of a large variety of consumer products in the fields of household care and personal care. We study the encapsulation ability of an amphiphilic poly(ethylene glycol)-*graft*-poly(vinyl acetate) (PEG-*g*-PVAc) graft copolymer, extending the focus to the entire phase diagram of polymer/perfume/water systems with three common natural fragrances. The three perfume molecules (2-phenyl ethanol, L-carvone, and α-pinene) possess different water affinities, as expressed by their octanol/water partition coefficients. The investigation of the polymorphism of PEG-*g*-PVAc in these systems is carried out by means of dynamic light scattering, small-angle X-ray scattering, NMR spectroscopy, and confocal laser scanning microscopy. The results presented here demonstrate that the choice of fragrance can dramatically affect the supramolecular structures formed by the polymer in aqueous solution, with important consequences on formulations of industrial interest such as the demixing of complex perfume blends when one or more of the components have no chemical affinity for any of the polymer blocks.

## Introduction

The encapsulation of poorly water-soluble compounds such as perfumes, flavors, and bioactive molecules is a key step in the formulation of a large variety of consumer products.^1^ A plethora of nano- and microencapsulation systems have been explored in the past decades to enhance the solubilization, protection, and controlled delivery of essential ingredients in various areas of the chemical industry.^2−4^ The fields of household care and personal care, where the encapsulation of fragrances and coloring agents is of paramount importance,^5,6^ are perhaps among the most demanding with respect to their performance requisites. Indeed, due to the complexity of typical matrices in cosmetics and cleaning agents, the formulation of valuable encapsulation systems entails a compromise of several qualities: good mechanical properties, stability (shelf life), controlled release, low toxicity of both precursors and finished product, biodegradability, cost-effective materials and processes, and scalable methods for industrial production.

Among all, laundry detergents and fabric enhancers face the growing need to meet regulatory requirements in environment-related legislative actions, as their release in wastewaters is unavoidable and the current materials employed in encapsulation technologies often present poor biodegradability profiles.^7^ Common substances for capsule wall production are amino resins like melamine–formaldehyde;^8,9^ alternatives have been explored since some time, such as polysulfones, chitosan, gum arabic, and maltodextrins, to name a few.^10−12^ However, the methods employed to drive the encapsulation of actives can be lengthy and difficult to upscale, especially when volatile molecules are involved, such as the use of microfluidics,^13^ layer-by-layer techniques,^14^ or solvent evaporation.^15^

**Scheme 1:**
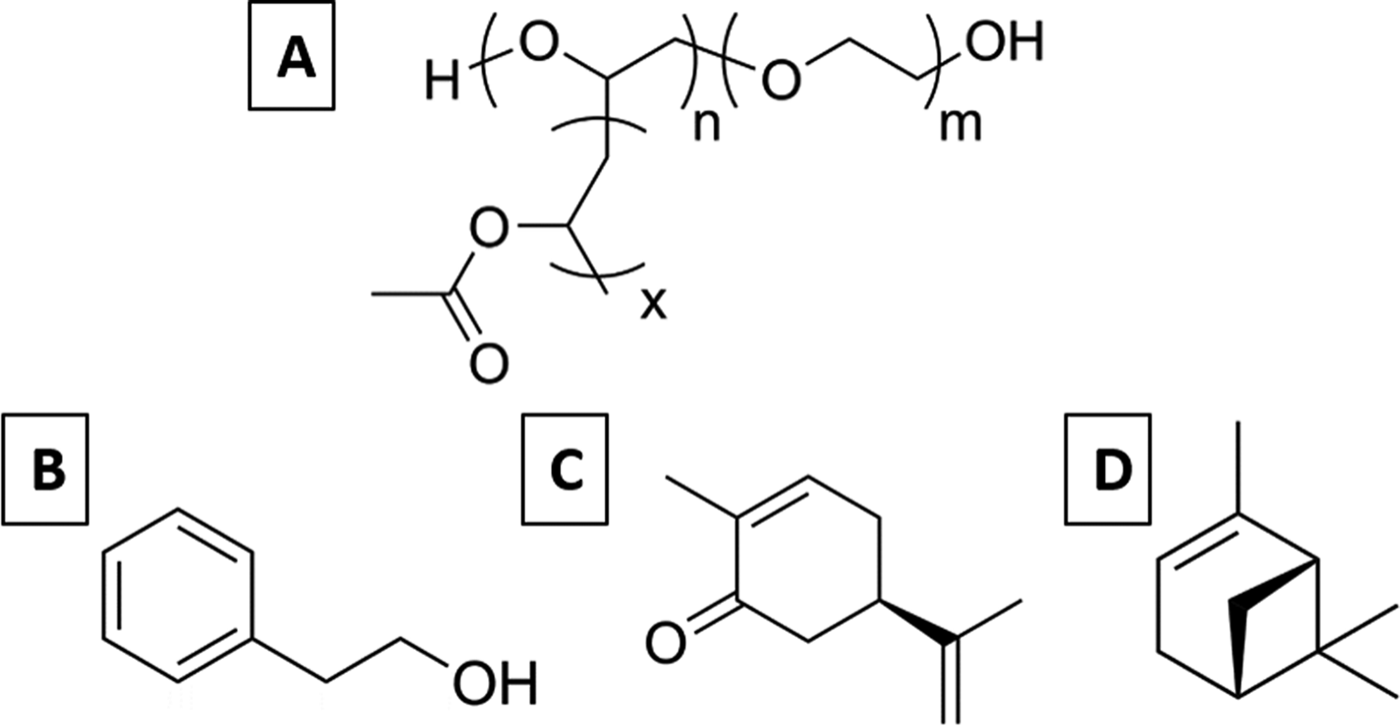
Molecular Structures of the Compounds Used in This Work (A) Poly(ethylene glycol)-*graft*-poly(vinyl acetate) (PEG-*g*-PVAc); (B) 2-phenyl ethanol (PE); (C) L-carvone (Car); and (D) (+)-α-pinene (Pin).

Nano- and microencapsulation systems such as micelles and liquid crystals based on nonionic block copolymers are typically very stable both thermodynamically and kinetically.^16,17^ Thanks to their solvent-selective blocks, these polymers are amphiphiles and they can form a whole range of supramolecular self-assembled structures common to small-molecule surfactants,^18^ which can be profoundly affected by the presence of encapsulated chemicals.^19^ It is therefore essential to understand the interactions existing between encapsulates and wall materials, including the exact location of fragrance molecules inside the polymer carrier, as these determine the formulation stability and the payload release.^16,20^ In this work, we focus on a poly(ethylene glycol)-*graft*-poly(vinyl acetate) (PEG-*g*-PVAc) copolymer with low grafting density (Scheme 1A), which has recently shown interesting properties as an encapsulating agent to protect hydrophobic compounds in aqueous solution^21^ and in detergent matrices.^22^ Thanks to its amphiphilic properties^23^ and its lower critical solution temperature (LCST) phase behavior, as well as its biodegradable blocks,^24−26^ this polymer is a very promising candidate as a perfume carrier for home care and personal care formulations.

In our previous investigation of dilute aqueous PEG-*g*-PVAc,^21^ we demonstrated the formation of globular single-chain nanoparticles (SCNPs) at a low polymer concentration (<10%) that could entrap fragrance molecules; dynamic light scattering (DLS) measurements showed that these unimeric micelles could be more or less swollen depending on the hydrophobicity of the encapsulate, according to Fischer’s model.^27^ In the present work, we extend our investigation to the phase behavior of the ternary systems in all possible concentration ratios, to elucidate the effect of the actives’ hydrophobicity and/or affinity for the polymer blocks on the polymorphism of PEG-*g*-PVAc. Clearly, knowledge of the ternary phase diagrams provides useful practical insights for consumer goods formulators, such as the correct dilution path to avoid the formation of excessively viscous liquid-crystal phases.^28^ Moreover, understanding the polymer behavior toward cosolvents and nonsolvents is of paramount importance to predict its properties as an emulsifier and formulation stabilizer, and to best apply it in everyday life products.^29−31^ We employ three simple natural fragrances characterized by molecular structures of similar bulkiness but different water affinities on account of their polarities, as expressed by their octanol/water partition coefficients (log *K*_ow_, sometimes referred to as log *P*):^32^ the hydrophobic α-pinene (“Pin”; log *K*_ow_ = 4.44), L-carvone (“Car”; log *K*_ow_ = 2.74, solubility in water = 0.4% w/v), and 2-phenyl ethanol (“PE”; log *K*_ow_ = 1.36, solubility in water = 2% w/v). These organic compounds are found in vegetal essential oils and commonly used in perfumery and personal care products (Scheme 1). The investigation of the polymorphism of PEG-*g*-PVAc in these systems is carried out by means of dynamic light scattering, small-angle X-ray scattering (SAXS), NMR spectroscopy, and confocal laser scanning microscopy.

## Materials and Methods

### Materials

Poly(ethylene glycol) (PEG; molecular weight (MW), 6 kDa), poly(vinyl acetate) (PVAc; MW, 7 kDa), and PEG-*g*-PVAc were products of BASF. PEG-*g*-PVAc is characterized by a PEG/VAc weight ratio of 40/60; *M*_n_ = 13.1 kDa, *M*_w_ = 27.5 kDa (polydispersity index (PDI) = 2.1), and a degree of branching of 1–2%.^21^ For confocal microscopy imaging purposes, PEG-*g*-PVAc was covalently labeled with rhodamine B isothiocyanate, according to a previously described procedure.^22^

The following reagents were purchased from Sigma-Aldrich (Milan, Italy) and used as received: L-carvone (Car, ≥97%, (FCC, FG), log *K*_ow_ = 2.74; MW, 150.22 g mol^–1^); 2-phenyl ethanol (PE, ≥99.0% (GC), log *K*_ow_ = 1.36; MW, 122.16 g mol^–1^); α-pinene (Pin, ≥99.0%, log *K*_ow_ = 4.44; MW, 136.23 g mol^–1^); rhodamine B isothiocyanate (mixed isomers; MW, 536.08 g mol^–1^); D_2_O (deuterium content >99%). Water used in this work was of Milli-Q grade (18.2 MΩ cm at 25 °C).

### Phase Diagrams

PEG-*g*-PVAc/PE/water, PEG-*g*-PVAc/Car/water, and PEG-*g*-PVAc/Pin/water ternary phase diagrams were constructed by weighing the appropriate amounts of water, polymer, and perfume in a glass vial with an analytical balance (Radwag AS R2; accuracy, ±0.1 mg); the polymer was molten at 50 °C for ease of manipulation. Samples were vortexed until homogenization using a standard VELP vortex mixer at a maximum speed of 3000 rpm, and they were stabilized at 25 °C in an oven for 14 days. Hereinafter, concentrations will always be expressed as weight percent unless specified differently. It is important to mention that the samples were kept in sealed vials and at a constant temperature in order not to affect the partition equilibrium of fragrance molecules between the liquid phase and the headspace, and thereby ensure a constant concentration in the formulations.

### Dynamic Light Scattering (DLS)

DLS measurements were performed on a Brookhaven BI9000-AT digital autocorrelator, equipped with a diode-pumped solid-state (DPSS) laser operating at λ = 532 nm (Torus, mpc3000, LaserQuantum, U.K.) and an avalanche photodiode (APD) detector positioned at 90°. Samples were placed in glass test tubes and immersed in a vat filled with decahydronaphtalene as a glass refraction index matching liquid. Experiments were performed at 25 °C; the temperature was controlled by a thermostatic bath with an accuracy of ±0.5 °C. Autocorrelation functions were analyzed via the cumulant method^33^ to extract the diffusion coefficients *D*, which were then converted into hydrodynamic radii, assuming a spherical shape, through the Stokes–Einstein equation
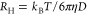
1where *R*_H_ is the hydrodynamic radius, *k*_B_ is the Boltzmann constant, and η is the viscosity of the solvent.

### Small-Angle X-ray Scattering (SAXS)

SAXS measurements were performed on a HECUS S3-MICRO camera equipped with a position-sensitive detector (OED 50 M) containing 1024 channels of width 54 μm. The X-ray source (GENIX-Fox 3D, Xenocs, Grenoble) operated at a maximum power of 50 W to provide an ultrabrilliant point microfocus Cu Kα radiation (wavelength λ = 1.542 Å). The sample-to-detector distance was 281 mm. SAXS curves were obtained in the *Q*-range between 0.009 and 0.54 Å^–1^ (where the modulus of the scattering vector is defined as *Q* = (4π/λ)sin θ, with 2θ the scattering angle). Samples were placed in either quartz Mark capillaries (liquids) or in a steel demountable cell using Kapton tape as windows (very viscous liquids or solids), and the cells were kept under vacuum during the experiments. All measurements were performed at the temperature 25 ± 0.1 °C (controlled by a Peltier element). All scattering curves were corrected for the empty cell contribution considering the relative transmission factor; data reduction and modeling were performed with the NIST package on the software IGOR Pro (WaveMetrics, Inc.)^34^ and with the software SasView.^35^

In a typical SAXS experiment, the scattered intensity *I*(*Q*) is a function of the scattering vector *Q*. For monodisperse centrosymmetric scattering objects
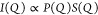
2where *P*(*Q*) is the form factor, related to the shape, size, and polydispersity of the scattering objects, and *S*(*Q*) is the structure factor, related to the interaction potential in the system.^36^ In very dilute noninteracting systems, *S*(*Q*) ∼ 1 and *I*(*Q*) ∝ *P*(*Q*). More information on SAXS data modeling can be found in the Supporting Information.

### NMR Spectroscopy

^1^H spectra and two-dimensional (2D) ^1^H–^1^H nuclear Overhauser enhancement spectroscopy (NOESY) correlation maps were recorded in D_2_O on a Bruker Avance 400 spectrometer, operating at a 400 MHz proton frequency, and using the peak of the solvent residual protons as internal reference. ^1^H–^1^H NOESY experiments were conducted with mixing times of 200 and 500 ms, 512 experiments in the F1 dimension with 16 scans for each of the increments on *t*_1_ and a sweep width of 15 ppm.

Self-diffusion experiments were performed using a Bruker Avance 300 MHz (7.05 T) spectrometer with an operating frequency of 300.131 MHz to perform ^1^H NMR experiments. Particularly, the spectrometer was equipped with a Bruker DIFF30 probe supplied by a Bruker Great 1/40 amplifier that can generate field gradients up to 1.2 T m. Measurements were carried out at 25 °C keeping the temperature constant (with an accuracy of 0.5 °C) by means of a BVT 3000 variable-temperature control unit. The pulse-gradient stimulated echo (PGSTE) sequence was used.^37^ Self-diffusion coefficients were obtained by varying the gradient strength (*g*) while keeping the gradient pulse length (δ) and the gradient pulse intervals constant within each experimental run. Data were fitted according to the Stejskal–Tanner equation
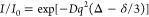
3where *I* and *I*_0_, respectively, are the NMR signals intensities in the presence or absence of the applied field gradient, *q* = γ*g*δ is the so-called scattering vector (γ being the gyromagnetic ratio of the observed nucleus), (Δ – δ/3) is the diffusion time, Δ is the delay time between the encoding/decoding gradients, and *D* is the self-diffusion coefficient to be extracted. When necessary, the diameter of nanoparticles was calculated with the Stokes–Einstein equation (eq 1) using the following solvent viscosities: 1.10 × 10^–3^, 7.58 × 10^–3^, and 2.61 × 10^–3^ Pa s, respectively, for D_2_O, PE, and Car.

### Confocal Scanning Laser Microscopy (CLSM)

CLSM imaging was carried out using a Leica TCS SP8 confocal microscope (Leica Microsystems GmbH, Wetzlar, Germany). Samples were placed in appropriate wells (Lab-Tek Chambered 1.0 Borosilicate Coverglass System, Nalge Nunc International, Rochester, NY). A 63× water immersion objective was used to image all samples. Rhodamine B (RhB) was excited at 561 nm with a DPSS laser, and the fluorescence emission was acquired using a hybrid SMD detector in the 571–600 nm range.

## Results and Discussion

To evaluate the possible behavior of the PEG-*g*-PVAc polymer in the presence of each perfume, we first performed solubility tests with the simple polymers PEG and PVAc (6 and 7 kDa, respectively). 2-Phenyl ethanol behaved as a good solvent for PEG and a poor solvent for PVAc; conversely, carvone was a poor solvent for PEG and a good solvent for PVAc; finally, pinene was a bad solvent for both. These are clear hints at the possible different miscibilities of the PEG-*g*-PVAc copolymer with each perfume molecule. Indeed, by mixing the graft copolymer with each fragrance compound in all concentration ratios from 10/90 to 90/10 wt %, we could determine that: (a) PE solubilizes PEG-*g*-PVAc up to 60 wt % polymer (for higher PEG-*g*-PVAc contents, a precipitate is formed); (b) polymer solubility does not reach above 30 wt % in carvone; and (c) there is no miscibility between the polymer and pure pinene at any ratio.

**Figure 1:**
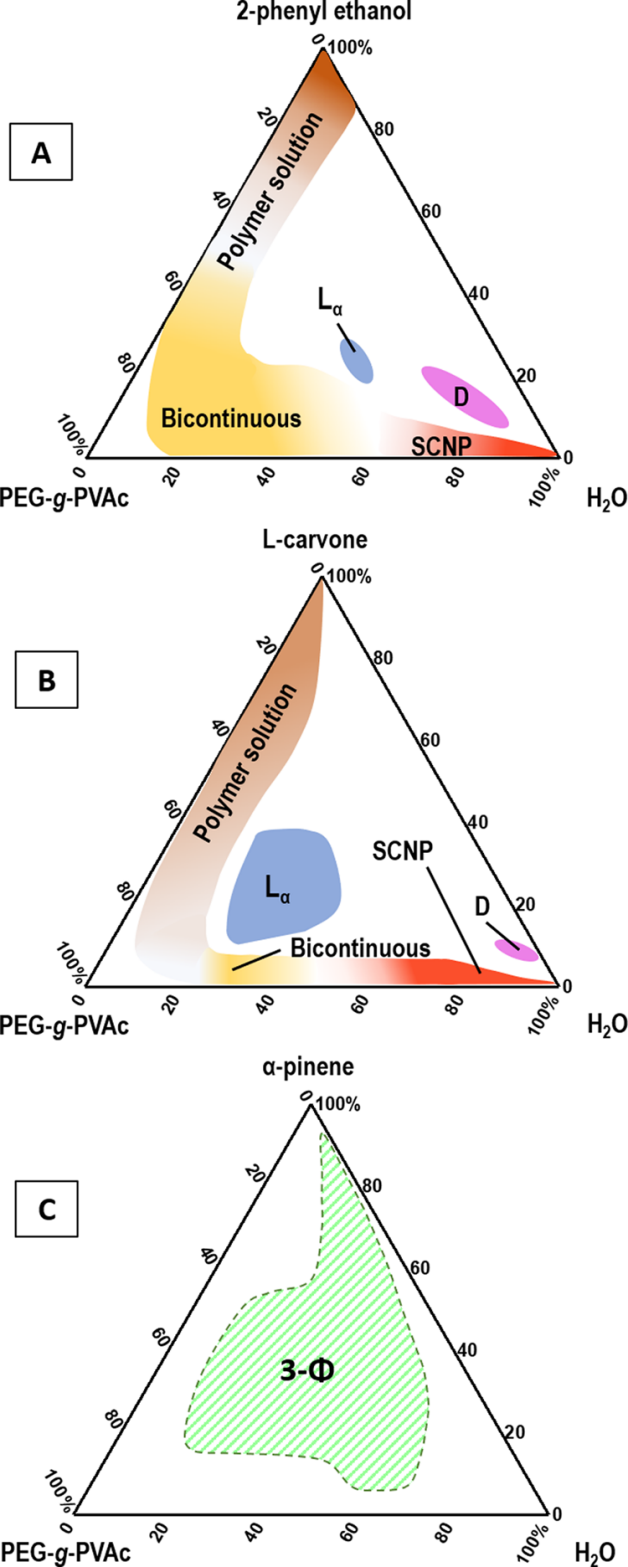
Gibbs ternary phase diagrams for the PEG-*g*-PVAc/perfume/water ternary systems at 25 °C: (A) 2-phenyl ethanol, (B) L-carvone, and (C) α-pinene. Concentrations are expressed in wt %. “SCNP” = single-chain nanoparticles; “D” = droplet phase; “L_α_” = lamellar mesophase; “3-Φ” = three-phase region. The white areas represent two-phase regions.

The latter results represent the binary polymer/perfume axes for each of the ternary diagrams we are about to investigate. The phase behavior in the binary PEG-*g*-PVAc/water axis is known,^38^ and so is the perfume/water miscibility for each fragrance compound. Figure 1 displays the Gibbs phase diagrams for the ternary systems PEG-*g*-PVAc/perfume/water (A = 2-phenyl ethanol; B = L-carvone; C = α-pinene) at 25 °C. As expected, the three plots are rather different. The PEG-*g*-PVAc/PE/water system is dominated by a continuous isotropic liquid region of varying viscosity, extending from the polymer/water binary axis toward the polymer/PE axis. Within this region, roughly three different substructures can be identified, which change from one to the other with no visible macroscopic interruption between subphases.

A similar region appears in the PEG-*g*-PVAc/Car/water diagram, but its extension is limited to a smaller area. Both phase diagrams present a more or less central region of viscous birefringent liquid, identified as a lamellar mesophase (“L_α_”), which is much larger for Car than for PE. Following a dilution line from the lamellar phase toward the H_2_O corner, in each diagram, a small region (“D”) of milky liquid exists, which is composed of micron-sized droplets. Finally, no single-phase areas were found in the PEG-*g*-PVAc/Pin/water system. We will now focus our discussion on the relevant regions of the diagrams, comparing the effects of the two different fragrances PE and Car on the PEG-*g*-PVAc phase behavior; the pinene phase diagram will be discussed last.

### PEG-*g*-PVAc/Perfume/Water Systems in the Highly Dilute Regime

**Figure 2:**
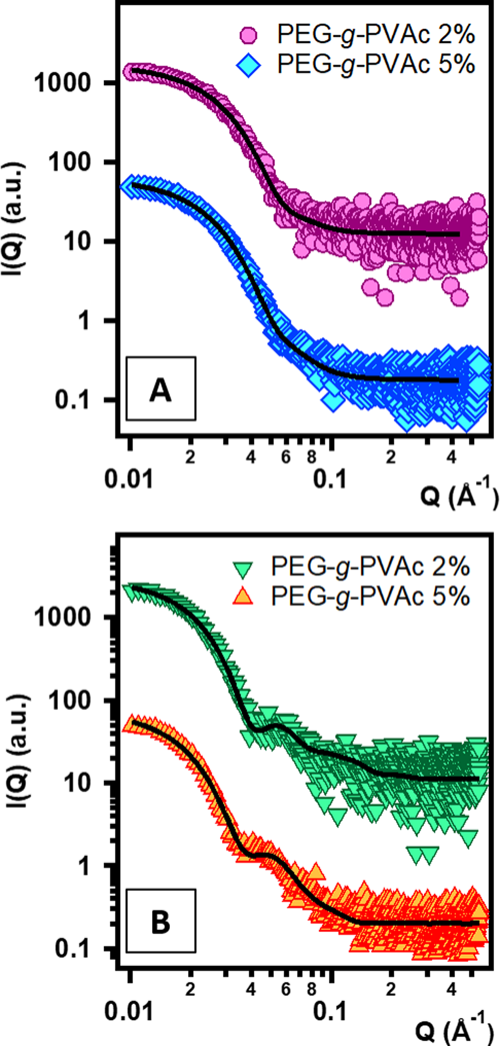
SAXS curves obtained for samples in the highly dilute regime of the PEG-*g*-PVAc/perfume/water ternary systems, with (A) 2-phenyl ethanol and (B) carvone. Polymer concentrations are indicated in the figure legends. Markers represent experimental points, while the solid lines represent the best fits to the models discussed in the text. Curves were offset along the *y* axis for presentation purposes.

Encapsulation systems for fast-moving consumer goods are often engineered for products that contain around 90% water, or reach similar water levels as they undergo dilution upon use. For this reason, it is interesting to begin our investigation of the ternary systems in the high-dilution regime. We prepared aqueous samples containing 2 and 5% polymer, with 0.5 and 1% perfume respectively, which were investigated by means of DLS (results are summarized in Table 1) and SAXS (results are shown in Figure 2 and Table 1).

**Table 1:** Results of DLS Measurements and SAXS Data Fitting for the Samples Evidenced in Figure 2^a^

sample composition	PEG-*g*-PVAc (wt %)	2.0	5.0	2.0	5.0
	2-phenyl ethanol (wt %)	0.5	1.0		
	L-carvone (wt %)			0.5	1.0
	water (wt %)	97.5	94.0	97.5	94.0
DLS	*R*_H_ (Å)	116	113	144	287
	PDI	0.06	0.10	0.13	0.26
SAXS—sphere form factor	*R* (Å)	66	71		
	σ	0.22	0.24		
	SLD_sphere_ (10^–6^ Å^–2^)	10.2	10.2		
SAXS—core–shell form factor	*R*_c_ (Å)			43	45
	σ			0.23	0.31
	SLD_core_ (10^–6^ Å^–2^)			8.99	8.99
	*t*_1_ (Å)			16	40
	SLD_s_1__ (10^–6^ Å^–2^)			10.5	10.5
	*t*_2_ (Å)			55	46
	SLD_s_2__ (10^–6^ Å^–2^)			9.51	9.51
	*R*_tot_^b^ = *R*_c_ + *t*_1_ + *t*_2_ (Å)			114	131

a DLS: *R*_H_ = hydrodynamic radius and PDI = polydispersity index, obtained from cumulant analysis of the autocorrelation functions. SAXS—sphere form factor: *R* = sphere radius; σ = Schulz polydispersity of *R*; SLD_sphere_ = scattering length density of the sphere. SAXS—core–shell form factor: *R*_c_ = core radius; σ = Schulz polydispersity of *R*; SLD_core_, SLD_s_1__, and SLD_s_2__ = scattering length densities of the core, first shell, and second shell, respectively; *t*_1_ and *t*_2_: thickness of the first and second shell, respectively; *R*_tot_: radius of the core–shell particle. Instrumental error associated with these results is ±0.6 Å.

b *R*_tot_ is not a fit model parameter.

DLS analysis of the samples containing 2-phenyl ethanol yielded hydrodynamic radii in close agreement with those we measured previously for the polymer single-chain nanoparticles (*R*_H_ = 112 Å for 2% neat polymer in water).^21^ The SAXS pattern of the system with 2% polymer and 0.5% perfume (Figure 2A) was well fitted with a function representing spheres (eq S1) having a Schulz distribution of the radii (eq S3): according to this treatment, the scattering objects in this solution are 66 Å in radius, roughly the same size found for the pure polymer single-chain nanoparticles (from here on, SCNP).^21^ The SLD of the spheres obtained from the fitting procedure was 10.2 × 10^–6^ Å^–2^, which is consistent with an average of the values for PEG, PVAc, and PE. This result indicates a complex mixing of the three species, suggesting the self-folding of the polymer into the SCNP structure embedding the perfume. Such structure does not vary when increasing the polymer content to 5% and the perfume to 1%.

In the presence of carvone, DLS measurements highlighted larger radii than those found in the PE samples. Also, the SAXS patterns in Figure 2B immediately appear different from the analogous PE curves. Indeed, the typical signature of a core–shell structure is present, and the experimental curves were fitted with a core–two-shell sphere form factor (eq S2). An SLD of 8.99 × 10^–6^ Å^–2^ was found for the core, which is close to the SLD of pure carvone (8.84 × 10^–6^ Å^–2^); the inner shell SLD, 10.5 × 10^–6^ Å^–2^, is consistent with the PVAc moiety, while the outer shell SLD, 9.51 × 10^–6^ Å^–2^, is consistent with highly hydrated PEG. DLS and SAXS results therefore suggest segregation of carvone in the SCNP core, leading to the swelling of the structure.

**Figure 3:**
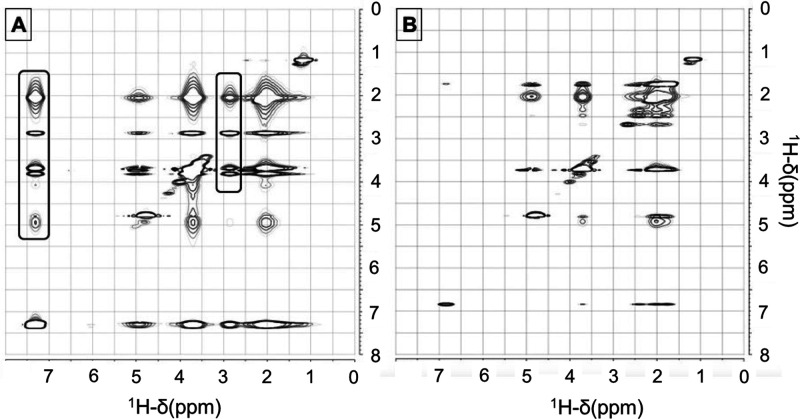
{^1^H–^1^H} NOESY correlation maps for (A) PEG-*g*-PVAc (5%)/PE (1%)/D_2_O and (B) PEG-*g*-PVAc (5%)/Car (1%)/D_2_O.

According to these results, both perfumes are encapsulated in the SCNP structure, but each seems to induce a different arrangement of the PEG and PVAc chains. To clarify this point, useful information about the nature of the interactions between PE and the polymer was obtained by 2D {^1^H–^1^H} NOESY correlation NMR experiments.^19,39,40^ Samples with 5% polymer and 1% perfume were prepared in D_2_O; we took care of performing SAXS experiments on these samples to confirm that replacing H_2_O with D_2_O did not significantly affect the self-assembled structures (see Figure S1, Supporting Information). As shown in Figure S2 (Supporting Information), the proton NMR spectrum of the PEG-*g*-PVAc/PE/water mixture presents a clear spectral signature for PE, with two well-resolved bands in the 7.25–7.40 and 2.80–2.90 ppm regions and a partly overlapped peak at 3.82 ppm. In addition, well-distinct resonances for the two blocks of the polymer can be found: the signal at 3.60 ppm being associated with the PEG segment and the bands in the 4.80–5.20 and 1.50–2.30 ppm regions due to different ^1^H nuclei of the PVAc portion. Interestingly, in-phase cross-peaks are present in the {^1^H–^1^H} NOESY correlation map (Figure 3) between all signals of PE and the resonances of both blocks of PEG-*g*-PVAc, as further indication that PE and the polymer are in very close contact one with the other, but no preferential interaction with PEG or PVAc portions seems to occur. This result reinforces the idea that 2-phenyl ethanol is embedded in the polymer matrix of the SCNP.

In the case of the PEG-*g*-PVAc/Car/water system, unfortunately, the main bands of carvone, in the 1.00–2.50 and 4.70–4.90 ppm regions of the spectrum (Figure S3, Supporting Information), almost completely overlap with the PVAc signals, thus severely limiting the possibility to be used as probes. Nonetheless, a single well-isolated resonance at 6.85 ppm, related with the =CH– proton of the carvone ring, is present and no clear cross-correlation of this signal with any band of the polymer is observable in the {^1^H–^1^H} NOESY map, thus suggesting no specific and strong interaction at play in this system. These observations could explain the preferential location of L-carvone in the core of the SCNP, leading to a situation that minimizes its interactions with either the water or the polymer phase.

This region was also investigated by means of PGSTE NMR experiments to extract the self-diffusion coefficients of the species along a dilution line in water extending from the H_2_O corner to about 30% polymer (see Figure S4, Supporting Information). Tables S1 and S2 report the self-diffusion coefficients of all of the diffusing species in the PEG-*g*-PVAc/PE/D_2_O and PEG-*g*-PVAc/Car/D_2_O systems, respectively. For all samples, the Stejskal–Tanner plot of the polymer was characterized by a biexponential decay, indicating the presence of two diffusing species. Let us start the description of the PE phase diagram from the binary sample at 5% polymer in water (“W95” in Figure S4 and Table S1): to take into account the contribution of interparticle collisions that reduces the nanoparticle diffusion and to obtain the self-diffusion coefficients free from obstruction effects *D*_0_, the equation *D*_ϕ_ = *D*_0_(1 – 2ϕ) was used, where ϕ is the SCNPs volume fraction (calculated assuming the density of the polymer equal to 1.2 g cm^–3^ on account of its PEG and PVAc blocks) and *D*_ϕ_ is the observed self-diffusion coefficient for the polymer.^41^ Then, using the Stokes–Einstein equation (eq 1), hydrodynamic radii of 25 and 155 Å were, respectively, calculated for the fast and slow diffusing species. The larger diameter is consistent with results from DLS; therefore, the slow component of the decay can be associated with the SCNPs’ diffusion, while the fast component suggests the presence of a polymer synthesis residual such as solvent traces or small oligomers, most likely of vinyl acetate.

The same treatment was applied to sample W90 (9% polymer, 1% PE), yielding a hydrodynamic radius of 321 Å, which is not reasonable for the SCNPs. This result is in agreement with previous work on the neat polymer/water system at similar concentration,^38^ evidencing interparticle interactions and, possibly, a modification of the particle morphology that results in the observed anomalous decrease in *D*_ϕ_. Moving along the sample series W90, W80, W70, an initial abrupt reduction (about 1 order of magnitude) of *D*_ϕ_ was detected, followed by a further, smoother decrease. Although less evident, a similar trend of the measured self-diffusion coefficients at increasing polymer concentration can be noted also for the fast component of the polymer diffusion. Differently, self-diffusion coefficients of PE and D_2_O follow an approximately linear decrease through the series. Results related to *D*_ϕ_ can be explained considering that, along this path, the system undergoes percolation at increasing polymer concentration, initiating with the formation of diffusing particles having a shape different from the spherical one, possibly elongated aggregates, closely interacting but not yet constituting a bicontinuous network. Results related to the other diffusing species can be rationalized in terms of the decreased volume available for the diffusion as the polymer concentration increases.

A similar analysis performed on the PEG-*g*-PVAc/Car/D_2_O system led to calculate, from the slow component of the polymer diffusion, a hydrodynamic radius for the diffusing particles equal to 303 Å for sample Z90 (9% polymer and 1% carvone), which compares well with the particle size found in the PE system. The evolution of the diffusion coefficients along the dilution line is an expression of the increasing interparticle interactions, similarly to the PE case.

### Evolution of the Polymer/Water Binary Axis with Added Perfume

**Figure 4:**
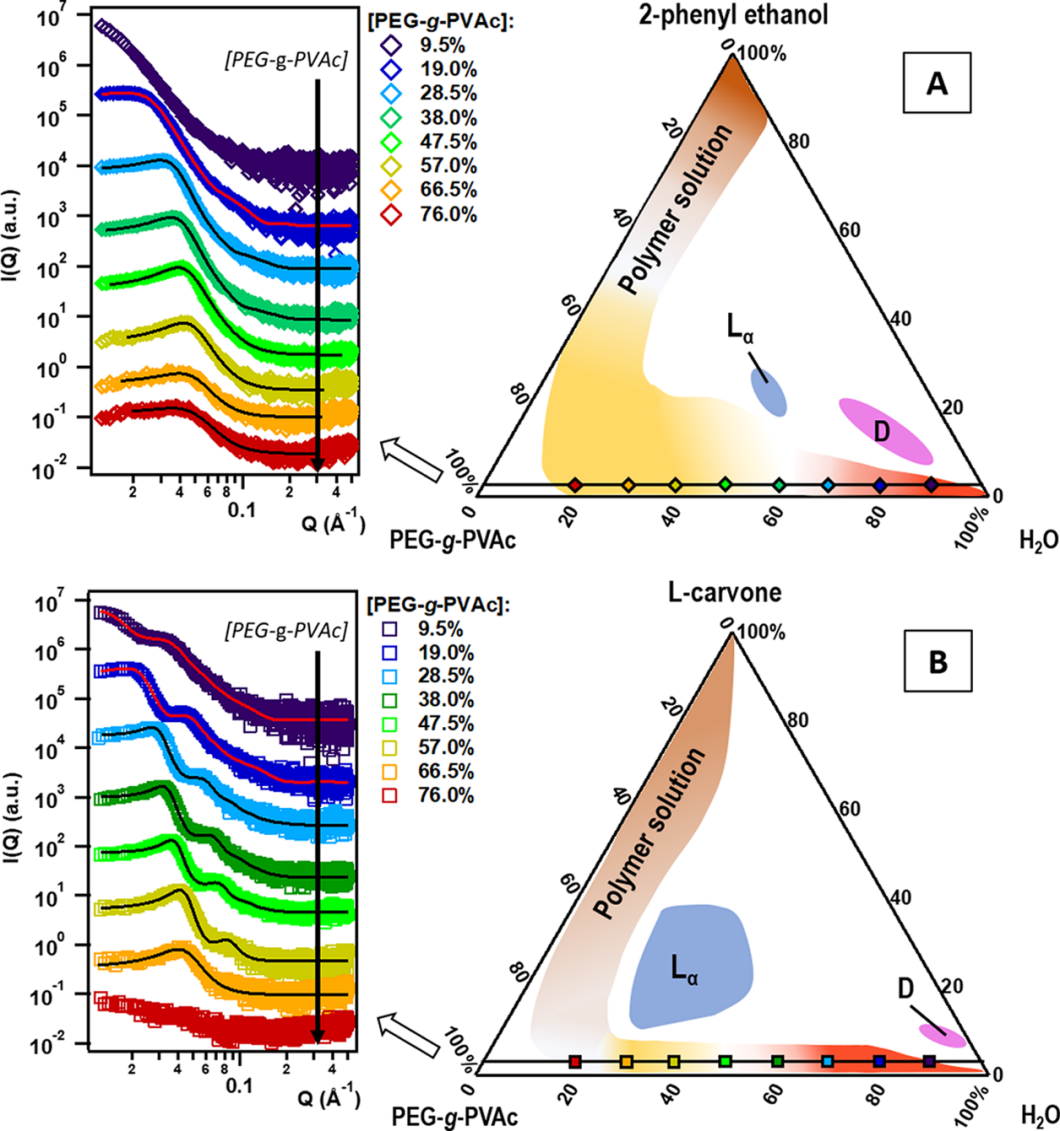
SAXS patterns obtained for the samples along the polymer/water axis, with 5% perfume added: (A) PE and (B) Car. Curves were offset along the *y* axis for presentation purposes.

Earlier investigation of the PEG-*g*-PVAc/water binary system^38^ evidenced that the SCNP formed at [PEG-*g*-PVAc] < 10% coexisted with an increasingly prominent bicontinuous network at higher polymer concentration, according to a percolative behavior. Here, we elucidate the effect of adding 5 wt % perfume to the binary system, using SAXS analysis. The curves obtained for these samples are evidenced in Figure 4. In the PE system, at 9.5% polymer content, a turbid sample is obtained, which splits into two phases in a matter of days. The scattering pattern suggests the presence of large aggregates (*d* > 100 nm; exact size cannot be determined as their Guinier region lies outside the available SAXS window). The cloud point of the polymer in this sample was measured by means of UV–vis spectroscopy (Figure S5, Supporting Information) and was found to be 5–7 °C; below such temperatures, the sample reverted to a single-phase clear solution. This suggests that, at 25 °C, larger aggregates are formed thanks to a decrease in the cloud point of PEG-*g*-PVAc caused by the interactions promoted by PE, similarly to what documented for this polymer in surfactant solutions.^22^

As the polymer concentration increases, the cloud point is raised above 25 °C and the system forms an isotropic solution with long-term colloidal stability that extends up to 80% PEG-*g*-PVAc. As we can observe in Figure 4A, SAXS curves for these samples present a characteristic correlation peak denoting a strong interaction between nearest-neighbor colloidal objects, in which they are reminiscent of those recorded for the binary system at similar PEG-*g*-PVAc concentrations.^38^ The peak position shifts to higher *Q* values with increasing polymer concentration, indicating a decrease in interparticle distance. In such conditions, it is difficult to separate the contribution of the form factor and the structure factor, and it can be risky to assume a shape for the aggregates. However, based on the results obtained earlier for the very dilute PEG-*g*-PVAc/PE SCNP in water, we can assume globular aggregates and model the curves according to a hard-sphere structure factor (eq S4).^42^ The high-*Q* part of the curves was better interpreted considering a core–shell form (eq S2 with a single-shell contribution with an SLD value of 1.08 × 10^–5^ Å^–2^ for the core and 9.51 × 10^–6^ Å^–2^ for the shell) rather than a full sphere. It is conceivable that, at such PEG-*g*-PVAc concentrations, the colloidal objects might no longer be single-chain nanoparticles, but rather micelle-like aggregates of two or more polymer chains where the PEG and PVAc blocks are better segregated into a hydrophobic core and a hydrophilic shell.

Fit results are summarized in Table S4 (Supporting Information): we observe that the total radius of the particles decreases with increasing polymer content (from 11.2 nm at 19.0% to 8.3 nm at 28.5%) due to the compression exerted by neighboring particles. At 38.0% polymer, the spherical model does not hold any longer, and from this point on, the patterns can be fitted using the Teubner–Strey model for bicontinuous structures (eq S6).^43^ These systems therefore consist of polymer physical networks, similarly to the neat PEG-*g*-PVAc/water systems.^38^ The coefficients obtained from the Teubner–Strey model allow for the calculation of the amphiphilicity factor, *f*_a_, which is a measure of the local order in an aqueous surfactant system (with the limits being *f*_a_ = −1 for the ordered lamellar liquid crystal and *f*_a_ = 1 for a disordered liquid).^44^ Here, we obtain negative values ranging from −0.3 to −0.5 for all samples: this shows that the presence of PE extends the persistence of stable bicontinuous polymer structures in a large area of the phase diagram and accounts for the evolution into a lamellar phase at a higher perfume content—although in a small region of the diagram.

In the analysis of the Car system, SAXS curves show an intense correlation peak moving to higher *Q* values as the polymer concentration increases, in the same way as we observed with PE. However, this main peak is followed by a number of bumps indicative of the core–shell signature as in the very dilute systems described earlier. These bumps also move to higher *Q* values with increasing PEG-*g*-PVAc content, and this qualitatively suggests a gradual compression of the cores and shells in the structures, possibly accompanied by interpenetration of the polymer coronas. We were able to model the SAXS patterns up to 57.0% polymer with a form factor representing core–two-shell spheres (eq S2); to correctly reproduce the low-*Q* peak, a hard-sphere structure factor (eq S4) was introduced, as done with the PE system. The results are reported in the Supporting Information (Table S5). Initially, the SLD of the core is the same as in the dilute core–shell systems, 8.9 × 10^–6^ Å^–2^, which is very close to the theoretical value for carvone; for a polymer content higher than 9.5%, however, we can obtain a good fit only by allowing the core SLD to increase gradually, which suggests a better homogenization of L-carvone among the PVAc chains. The modeling confirmed that the core radius and the PEG layer thickness decrease (the former from 87 to 48 Å; the latter from 43 to 8 Å), due to the compression originating from the hard-sphere interaction between particles.

It must be noted that, for effective volume fractions higher than 0.35, the hard-sphere volume fractions found upon modeling of the SAXS patterns were lower than expected. The fact that the hard-sphere potential does not hold at such high volume fractions is a characteristic property of soft particles.^45^ For the samples at [PEG-*g*-PVAc] = 57.0 and 66.5%, the curves were modeled using the Teubner–Strey function (eq S6), which yielded negative amphiphilicity factors around −0.5 similarly to the PE case.

### Lamellar Mesophase and “D” Regions

**Figure 5:**
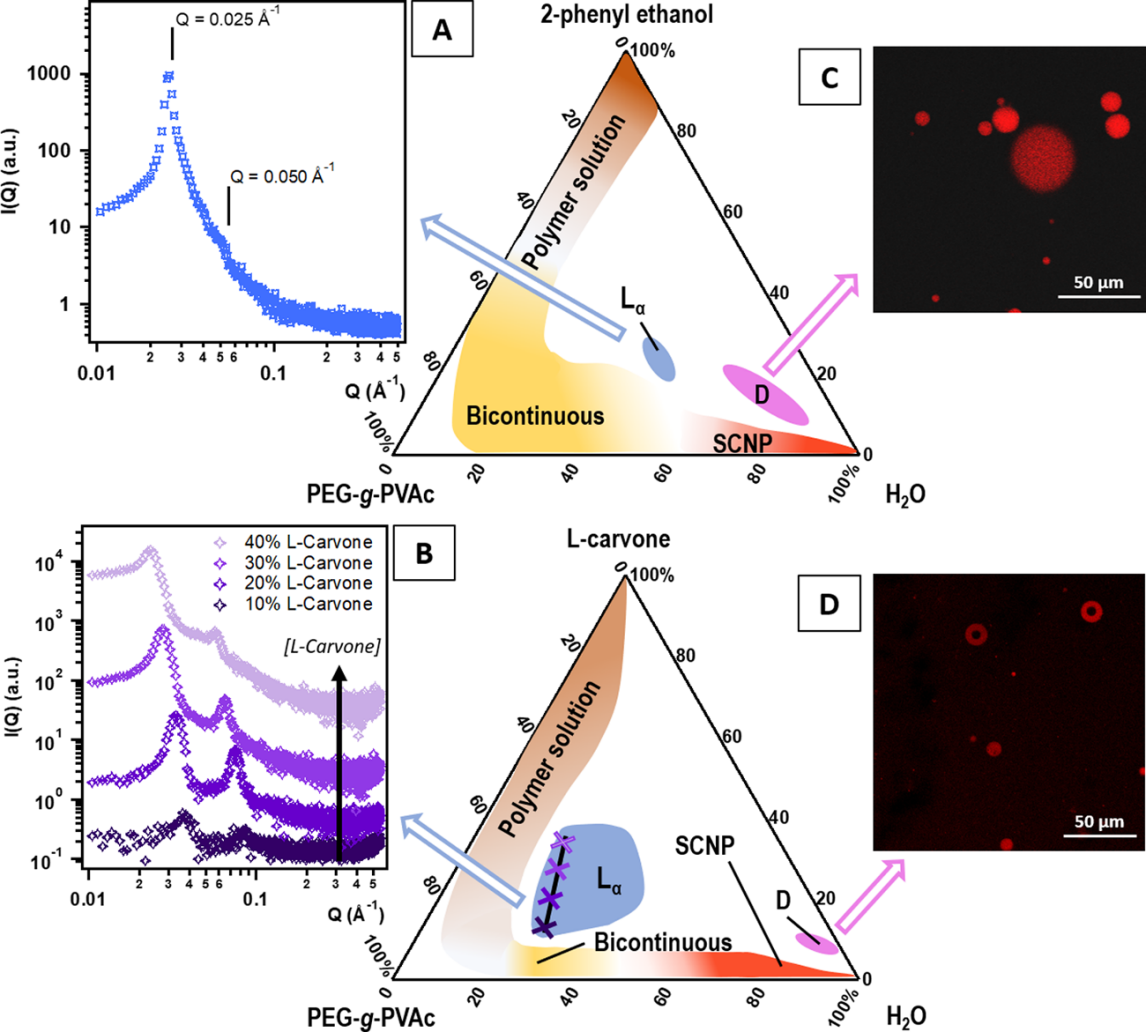
(A, B) SAXS patterns obtained for: (A) a representative sample from the L_α_ region in the PEG-*g*-PVAc/2-phenyl ethanol/water system (28/30/42 wt %) and (B) four samples along a dilution line in perfume in the L_α_ region of the PEG-*g*-PVAc/L-carvone/water system (curves were offset along the *y* axis for presentation purposes). (C, D) Confocal scanning laser microscopy images of representative samples in the “D” region of the phase diagrams of (C) PEG-*g*-PVAc/2-phenyl ethanol/water and (D) PEG-*g*-PVAc/L-carvone/water.

At intermediate polymer concentrations, the presence of 2-phenyl ethanol at around 25 wt % leads to a transition from the disordered bicontinuous structure to an ordered lamellar phase. The double-layer liquid crystal was identified by means of polarized-light optical microscopy (not shown) as well as SAXS experiments (Figure 5A); the latter evidenced typical Bragg peaks following the *Q*-sequence 1:2. The interlamellar distance (*d*) was calculated according to the relation 2π*n*/*Q*(*n*), where *n* is the order of diffraction and *Q*(*n*) is the corresponding *Q* value,^18^ yielding *d* = 250 Å for a representative sample taken in the center of the L_α_ region.

In contrast, in the PEG-*g*-PVAc/Car/water phase diagram, a larger area is covered by a region of lamellar liquid crystals, which is shifted to higher polymer concentration. The SAXS analysis of four samples within this area (Figure 5B), selected along a carvone dilution line, allowed to calculate the lattice spacings shown in Table 2. These results prove that the L_α_ structure is swollen as the concentration of the perfume increases from 10% (*d* = 165 Å) to 40% (*d* = 273 Å), suggesting the insertion of carvone in the hydrophobic PVAc palisade. An opposite trend has been observed in Pluronic lamellar phases with increasing content of hydrophobic fragrances,^46^ probably due to a different organization of the supramolecular assembly in the mesophase (and, consequently, different swelling behavior) determined by the architectures of the copolymers (*i.e*., comb-like *vs* linear).

**Table 2:** Lamellar Lattice Spacing Parameters (*d*) Corresponding to Four Samples along a Carvone Dilution Line (Polymer/Water Ratio Held Constant at 70/30 wt %)

L-carvone content (wt %)	*d* (Å, ±0.6 Å)
10	165
20	190
30	232
40	273

Moving from the L_α_ regions along a dilution line toward the water corner, we encounter the final regions of interest in each phase diagram, termed “D”, in which a typical sample appears as a milky white liquid. Observation with the optical microscope revealed spherical objects in the tens of microns size range, suggesting the spontaneous (or rather low-energy) formation of a remarkably stable emulsion. To shed more light into the aggregates’ structure, the same sample was prepared using rhodamine B-labeled PEG-*g*-PVAc, and observed by means of confocal scanning laser microscopy: the micrograph (Figure 5C) shows spherical objects of heterogeneous sizes, ranging from a few microns to about 40 μm. Scanning one of these spheres along the *z* axis reveals that the red fluorescence is homogeneously distributed within the object. This polymer is known to form similar structures in the presence of surfactant mixtures,^22^ which have been identified as microsegregated coacervates deriving from liquid–liquid phase separation. This suggests that PE could drive the liquid–liquid phase separation by lowering the cloud point of the polymer, and it should therefore be embedded in the polymer matrix.

The droplet region also exists in the presence of L-carvone, but its extension is reduced, and its position is shifted closer to the water corner. Fluorescently labeled PEG-*g*-PVAc samples investigated under the confocal microscope (Figure 5D) showed, surprisingly, a different structure for the phase-separated droplets: instead of full polymer spheres, carvone induces the formation of structures resembling giant polymersomes with an average radius of around 15 μm and a polymer shell thickness of around 4 μm. Interestingly, the structure of these aggregates seems to follow hierarchically the structure of the SCNP at higher dilution: full spheres for PE and core–shell for Car.

The milky suspensions appeared to form spontaneously upon gentle mixing of the three components, and they were stable for long periods of time (at least 6 months). They resisted centrifugation, freezing, and heating up to 50 °C. However, the real thermodynamic stability of such systems cannot be confirmed by these properties alone; this aspect deserves further study and will be dealt with in future work. For the present scope, it is worth mentioning that physicochemical coacervation or phase separation of the encapsulating material with the core compound (triggered by temperature changes, salting out, addition of nonsolvent) is used as an encapsulation method in some applications.^17^ These considerations support the possibility to employ fragrance-driven coacervation of PEG-*g*-PVAc aqueous dispersions as a robust encapsulation method in products containing high levels of water.

### PEG-*g*-PVAc/α-Pinene/Water System

**Figure 6:**
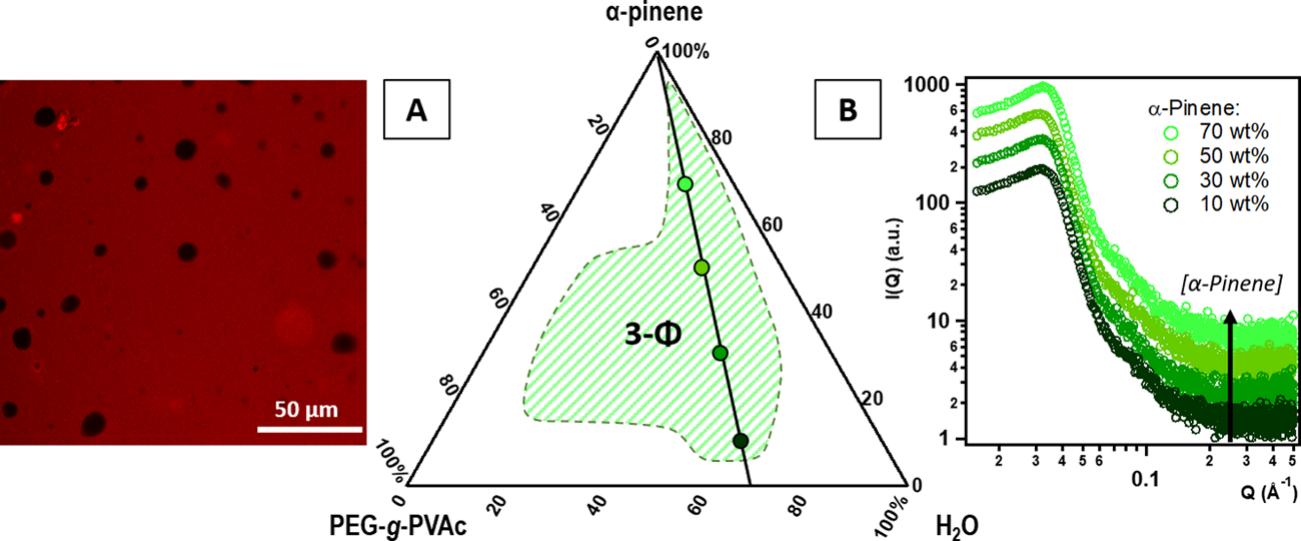
PEG-*g*-PVAc/α-pinene/water phase diagram at 25 °C; the green-shaded area indicates a three-phase region, and the white areas indicate two-phase regions. (A) CLSM micrograph obtained for the middle phase of a typical sample in the 3-Φ region, prepared with RhB-labeled polymer; (B) SAXS patterns obtained for the middle phases of samples along the dilution line characterized by a polymer/water ratio = 30/70 wt %, with increasing α-pinene concentration, as evidenced in the phase diagram. Curves were offset along the *y* axis for presentation purposes.

The phase diagram for the ternary system PEG-*g*-PVAc/α-pinene/water at 25 °C, shown in Figure 6, is dominated by a three-phase region. In a typical sample, the upper and lower phases are isotropic, while the middle one is opaque. This is remindful of a Winsor-III-type microemulsion,^47^ where a central phase rich in surfactant (bicontinuous microemulsion) is in equilibrium with excess water and oil (lower and upper phases, respectively, considering the densities). This suggests that very low amounts of pinene might actually be miscible with the polymer.

CLSM imaging of the middle phase (Figure 6A), from samples prepared with rhodamine B-labeled polymer, revealed diffuse fluorescence from a concentrated aqueous polymer phase dotted with black spherical objects; the latter are most likely droplets of insoluble perfume trapped in the polymer phase due to its high viscosity. SAXS curves (Figure 6B) of the middle phase in four samples, taken along a dilution line in pinene covering almost the entire phase diagram, are almost superimposable: this shows that the nanostructure depends on the polymer/water ratio, which does not vary. The scattering patterns are very similar to the ones obtained for samples in the PE and Car systems at a high polymer concentration, consistent with a bicontinuous structure.

## Conclusions

The selective solubilization of organic compounds by block copolymer micelles has been known since Nagarajan’s work,^48^ and many studies have explored the link between a fragrance’s log *K*_ow_ value and its preferential location in a micellar structure.^27^ The situation is especially complex in the intermediate hydrophobicity range, between log *K*_ow_ 2 and 3.5, where the effects of molecular structure on the favored partitioning locus become preponderant over simple hydrophobicity considerations.^49,50^ One drawback of many studies, however, is to limit the investigation to the extremely dilute micellar phases and to assume, for the micelles, completely segregated core and shell regions consisting of each of the polymer blocks in consideration.^51^ In this work, we have extended the focus to the entire phase diagram of polymer/perfume/water systems using the amphiphilic PEG-*g*-PVAc and three common natural fragrances used in perfumery. The results presented here demonstrate that the choice of fragrance can dramatically affect the supramolecular structures formed by this polymer in aqueous solution. We have shown that 2-phenyl ethanol and L-carvone are both encapsulated in polymer single-chain nanoparticles, while α-pinene is too hydrophobic and it separates from the self-assembled structures at all ratios. Moreover, the two successfully encapsulated fragrances lead to similar phase behaviors but different nanostructures (matrix-like for PE and core–shell for Car), and the borders of the thermodynamically stable regions differ in the two-phase diagrams. One of the possible consequences on final formulations could be the demixing of complex perfume blends when one or more of the components have no affinity for any of the polymer blocks. In conclusion, the graft copolymer PEG-*g*-PVAc described here is an extremely promising candidate for the encapsulation of actives in a number of different applications. Thanks to its varied polymorphism, it offers a choice of thermodynamically stable means of encapsulation, where the spontaneous formation upon simple mixing of the components results in a low-energy input necessary for production and thus cost-effective production, as long as the formulation design takes into account the details of encapsulate-to-polymer blocks interactions.

## Supporting Information Available

SAXS curves of dilute systems; NMR spectra; results of NMR diffusion experiments; and details on SAXS modeling and fit results (PDF)
