# Supplementary material for: Tuning the Encapsulation of Simple Fragrances with an Amphiphilic Graft Copolymer
Source: ACS Appl Mater Interfaces. 2020 May 28;12(25):28808–18. doi: 10.1021/acsami.0c05892 (PMC8007072; doi:10.1021/acsami.0c05892)
Supplement: Supplementary file 1 — am0c05892_si_001.pdf [file am0c05892_si_001.pdf]

Supporting Information

Tuning the encapsulation of simple fragrances with an amphiphilic graft copolymer

Marianna Mamusa,[1] Constantina Sofroniou,[1] Claudio Resta,[1] Sergio Murgia,[2] Emiliano Fratini,[1] Johan Smets,[3] Piero Baglioni[1*]*

[1]*Department of Chemistry "Ugo Schiff" and CSGI, University of Florence, Via della Lastruccia 3, Sesto Fiorentino, 50019 Florence, Italy*

[2]*Dipartimento di Scienze Chimiche e Geologiche, Università degli Studi di Cagliari, S.S. 554 Bivio per Sestu, 09042 Monserrato (CA), Italy*

[3]*The Procter & Gamble Company, Temselaan 100, 1853 Strombeek Bever, Belgium*

*Corresponding Authors

E-mail: mamusa@csgi.unifi.it; baglioni@csgi.unifi.it

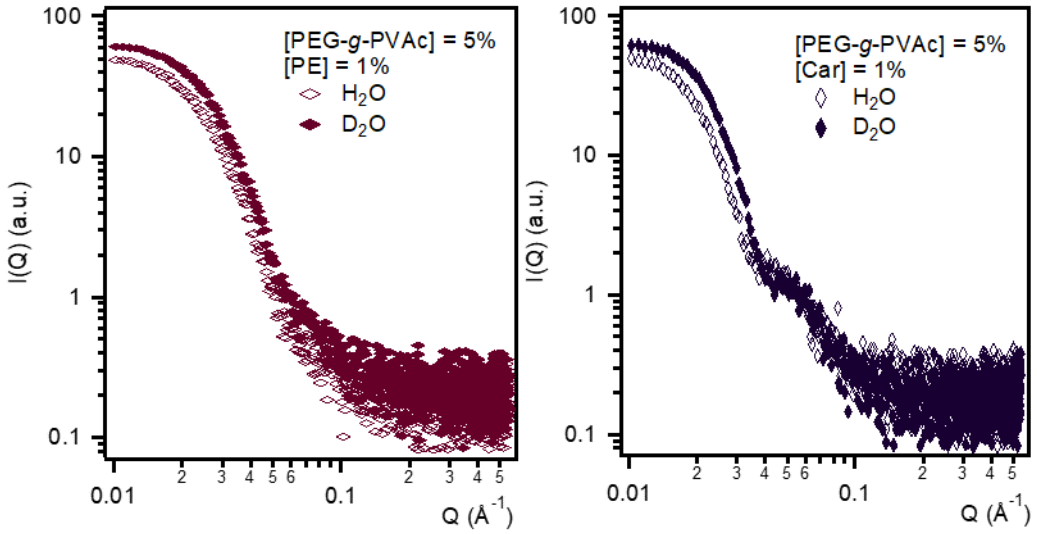

Figure S1. Comparison of SAXS curves for dilute PEG-*g*-PVAc/PE/water (left) and PEG-*g*-PVAc/Car/water (right) systems in normal water and heavy water.

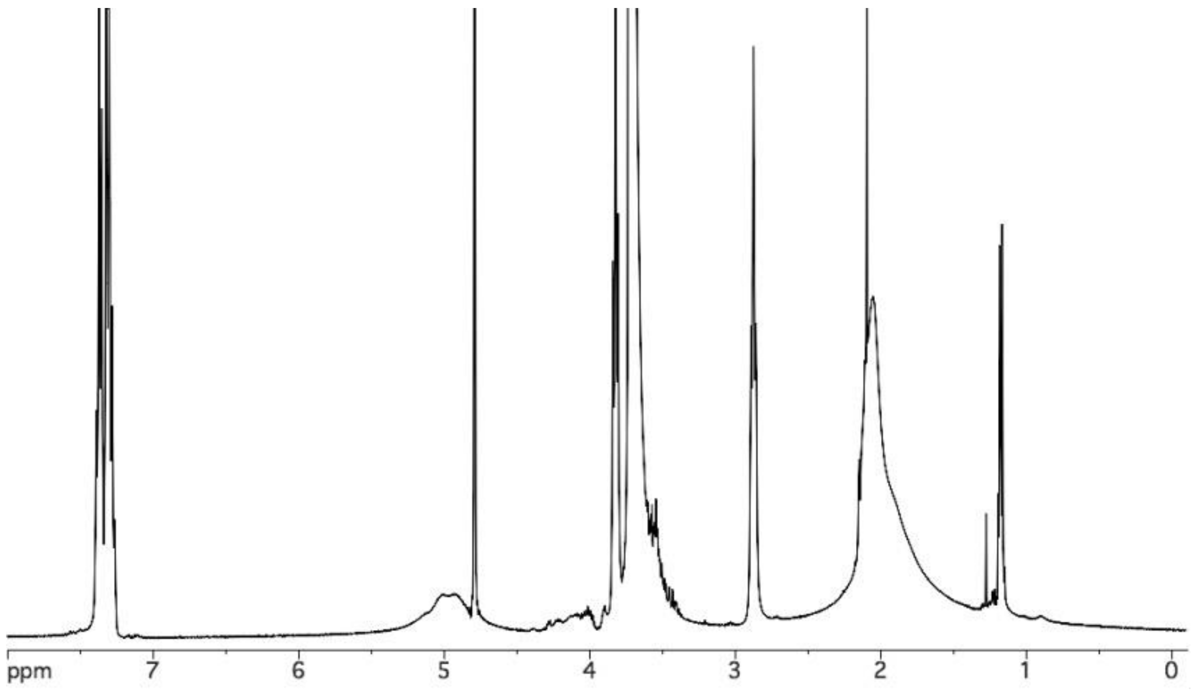

Figure S2: PEG-*g*-PVAc (5%)/2-phenylethanol (1%)/D_2O ^{1}H-NMR spectrum.

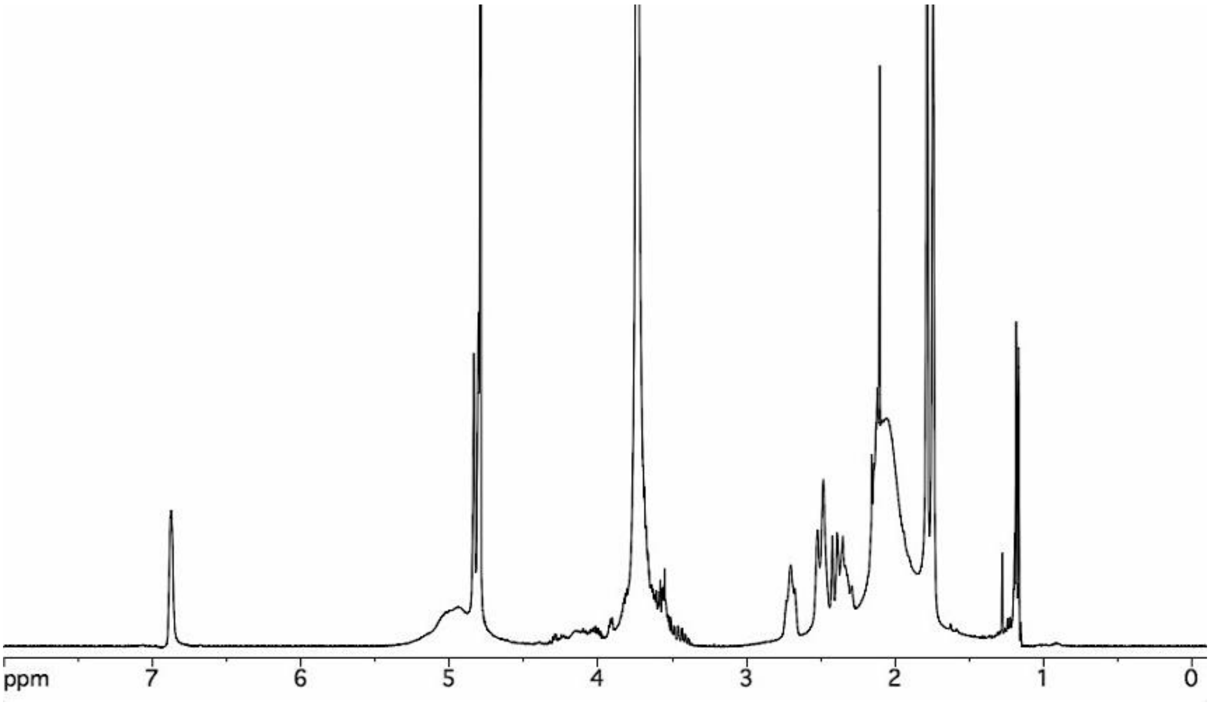

Figure S3: PEG-*g*-PVAc (5%)/Carvone (1%)/D_2O ^{1}H-NMR spectrum.

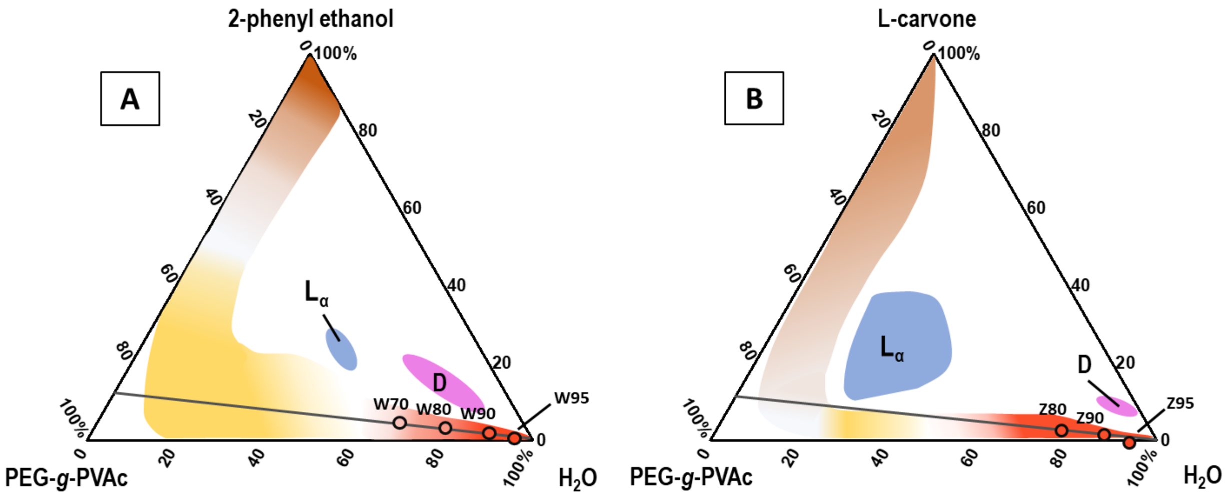

Figure S4. Positions of the samples investigated by means of NMR self-diffusion measurements in the PEG-*g*-PVAc/perfume/water phase diagrams with A) 2-phenyl ethanol and B) L-carvone.

Table S1. Compositions and extracted diffusion components for samples investigated by PGSTE NMR.

Sample name	Composition (%wt)			Self-Diffusion Coefficients, D_φ (m^2/s)			
	PE	D_2O	Polymer	PE	D_2O	Polymer	
						Fast	Slow
W90	1	90	9	$2.13\ 10^{-10}$	$1.53\ 10^{-09}$	$5.59\ 10^{-11}$	$5.26\ 10^{-12}$
W80	2	80	18	$1.16\ 10^{-10}$	$1.27\ 10^{-09}$	$2.11\ 10^{-11}$	$5.52\ 10^{-13}$
W70	3	70	27	$6.82\ 10^{-11}$	$9.73\ 10^{-10}$	$1.15\ 10^{-11}$	$3.33\ 10^{-13}$
**Controls**	**PE**	**D_2O**	**Polymer**	**PE**	**D_2O**	**Polymer**	
						**Fast**	**Slow**
PE/D_2O	1	99	-	$6.75\ 10^{-10}$	-	-	-
W95	-	95	5	-	$1.76\ 10^{-09}$	$7.35\ 10^{-11}$	$1.29\ 10^{-11}$

Table S2: Compositions and extracted diffusion components for samples investigated by PGSTE NMR.

Sample name	Composition (%wt)			Self-Diffusion Coefficients, D_φ (m^2/s)			
	Car	D_2O	Polymer	Car	D_2O	Polymer	
						Fast	Slow
Z90	1	90	9	$3.60\ 10^{-10}$	$1.63\ 10^{-09}$	$5.50\ 10^{-11}$	$5.57\ 10^{-12}$
Z80	2	80	18	$2.63\ 10^{-10}$	$1.32\ 10^{-09}$	$3.06\ 10^{-11}$	$1.08\ 10^{-12}$
**Controls**	**Car**	**D_2O**	**Polymer**	**Car**	**D_2O**	**Polymer**	
						**Fast**	**Slow**
Car/D_2O	1	99	-	$6.68\ 10^{-10}$	-	-	-
Z95	-	95	5	-	$1.76\ 10^{-09}$	$7.35\ 10^{-11}$	$1.29\ 10^{-11}$

Modelling of SAXS data

The following *P(Q)* functions were used to fit the SAXS experimental data in this work:

-Form factor for spherical objects:

$$P(Q) = \frac{A}{V}\left[\frac{3V(\Delta\rho)(\sin(QR) - QR\cos(QR))}{(QR)^3}\right]^2 + bkg \qquad \text{(S1)}$$

Here *A* is a scale factor, *V* and *R* are the volume and radius, respectively, of the scattering objects, $\Delta\rho$ is the so-called contrast i.e. the difference between the scattering length densities (SLDs) of the particles and the solvent, and bkg is the instrumental background signal.

-Form factor for core-shell spheres (with 2 shells):

$$P(Q) = \frac{A}{V_s}\left[\frac{3V_c(\rho_c - \rho_{s1})j_1(QR_c)}{QR_c} + \frac{3V_{s1}(\rho_{s1} - \rho_{s2})j_1(QR_{s1})}{QR_{s1}} + \frac{3V_{s2}(\rho_{s2} - \rho_{solv})j_1(QR_{s2})}{QR_{s2}}\right]^2 + bkg \qquad \text{(S2)}$$

Here the subscripts have the following meanings: *c* = core, s_1 = first shell, s_2 = second shell, *solv* = solvent; ρ is the SLD; $j_1(x) = (\sin x - x\cos x)/x^2$; $R_{s1} = R_c + t_1$ and $R_{s2} = R_c + t_1 + t_2$, with *t* the thickness of each shell; $V_i = (4\pi/3)R_i^3$.

Wherever relevant, a Schulz distribution of the radii was used:

$$f(R) = (z+1)^{z+1}x^z\frac{exp[-(z+1)x]}{R_{avg}\Gamma(z+1)} \qquad \text{(S3)}$$

where $z = 1/(PDI^2 - 1)$ is a function of the polydispersity, $PDI = \sigma/R_{avg}$, with σ^2 the variance of the distribution, R_{avg} the mean radius, $x = R/R_{avg}$ and Γ the Gamma function.

The interaction between polymer aggregates was interpreted according to a hard-sphere structure factor and the Percus-Yevick closure to the Ornstein-Zernike equation:[1,2]

$$S(Q) = \left[1 + \frac{24\phi_{HS}G(2QR_{HS})}{2QR_{HS}}\right]^{-1} \qquad \text{(S4)}$$

where $G(2QR_{HS})$ is a trigonometric function depending on Q, R_{HS} (hard-sphere radius) and ϕ_{HS} (hard spheres volume fraction). The potential has the form:

$$U(r) = \begin{cases} \infty & r < 2R \\ 0 & r \geq 2R \end{cases} \tag{S5}$$

For systems in the high polymer concentration regime, the Teubner-Strey model was used:[3]

$$I(Q) = \frac{1}{a_2 + c_1 Q^2 + c_2 Q^4} + bkg \tag{S6}$$

The coefficients a, c_1, and c_2 allow for calculation of the bicontinuous network's lattice size, or persistence length, ξ, and the repeat distance, d, through:[4,5]

$$\frac{d}{2\pi} = \left[\frac{1}{2}\left(\frac{a_2}{c_2}\right)^{1/2} - \frac{c_1}{4c_2}\right]^{-1/2} \tag{S7}$$

$$\xi = \left[\frac{1}{2}\left(\frac{a_2}{c_2}\right)^{1/2} + \frac{c_1}{4c_2}\right]^{-1/2} \tag{S8}$$

From these, the amphiphilicity factor, f_a, is calculated:[4]

$$f_a = \frac{1 - \left(\frac{2\pi\xi}{d}\right)}{1 + \left(\frac{2\pi\xi}{d}\right)} \tag{S9}$$

In our data modelling, we used the following SLD values for the chemical species in the systems:

Table S3. X-ray (Cu K_α) SLD values calculated for the compounds used in the present work.

Compound	Molecular formula	SLD (Å^{-2})
PEG	C_2H_4O	1.10 10^{-5}
PVAc	$C_4H_6O_2$	1.08 10^{-5}
Water	H_2O	9.36 10^{-6}
2-phenyl ethanol	$C_8H_{10}O$	9.29 10^{-6}
L-carvone	$C_{10}H_{14}O$	8.84 10^{-6}
α-pinene	$C_{10}H_{16}$	8.07 10^{-6}

Table S4. Fitting results for the SAXS patterns of Figure 4A (main text). R_c = core radius; σ = Schulz polydispersity of R; SLD_{core}, SLD_s = scattering length densities of the core and shell, respectively; t = shell thickness; R_{tot} = radius of the core-shell particle; Φ = hard-sphere volume fraction. Instrumental error associated to these results is ± 0.6 Å. ‡R_{tot} is not a fit model parameter.

<table>
<tr><td rowspan="3">Samples composition</td><td>PEG-g-PVAc (wt%)</td><td>19.0</td><td>28.5</td><td colspan="2">38.0</td><td>47.5</td><td>57.0</td><td>66.5</td><td>76.0</td></tr>
<tr><td>2-phenyl ethanol (wt%)</td><td>5.0</td><td>5.0</td><td colspan="2">5.0</td><td>5.0</td><td>5.0</td><td>5.0</td><td>5.0</td></tr>
<tr><td>Water (wt%)</td><td>76.0</td><td>66.5</td><td colspan="2">57.0</td><td>47.5</td><td>38.0</td><td>28.5</td><td>19.0</td></tr>
<tr><td rowspan="7">Core-shell form factor + hard-sphere structure factor</td><td>R_c (Å)</td><td>48 ± 0.3</td><td>37 ± 0.2</td><td>35 ± 0.2</td><td>-</td><td>-</td><td>-</td><td>-</td><td>-</td></tr>
<tr><td>σ</td><td>0.30</td><td>0.38</td><td>0.30</td><td>-</td><td>-</td><td>-</td><td>-</td><td>-</td></tr>
<tr><td>SLD_{core} (10^{-6} Å^{-2})</td><td>10.8</td><td>10.8</td><td>10.8</td><td>-</td><td>-</td><td>-</td><td>-</td><td>-</td></tr>
<tr><td>t (Å)</td><td>64 ± 0.2</td><td>46 ± 0.3</td><td>40 ± 0.3</td><td>-</td><td>-</td><td>-</td><td>-</td><td>-</td></tr>
<tr><td>SLD_s (10^{-6} Å^{-2})</td><td>9.51</td><td>9.51</td><td>9.51</td><td>-</td><td>-</td><td>-</td><td>-</td><td>-</td></tr>
<tr><td>‡$R_{tot} = R_c + t$ (Å)</td><td>112</td><td>83</td><td>75</td><td>-</td><td>-</td><td>-</td><td>-</td><td>-</td></tr>
<tr><td>Φ</td><td>0.14</td><td>0.22</td><td>0.23</td><td>-</td><td>-</td><td>-</td><td>-</td><td>-</td></tr>
<tr><td rowspan="3">Teubner-Strey model</td><td>Correlation length, ξ (Å)</td><td>-</td><td>-</td><td>-</td><td>73</td><td>70</td><td>63</td><td>50</td><td>42</td></tr>
<tr><td>Repeat distance, d (Å)</td><td>-</td><td>-</td><td>-</td><td>172</td><td>152</td><td>140</td><td>143</td><td>148</td></tr>
<tr><td>Amphiphilicity factor, f_a</td><td>-</td><td>-</td><td>-</td><td>-0.5</td><td>-0.5</td><td>-0.5</td><td>-0.4</td><td>-0.3</td></tr>
</table>

Table S5. Fitting results for the SAXS patterns shown in Figure 4B (main text). R_c: core radius; σ: Schulz polydispersity of R; SLD_{core}, SLD_{s1} SLD_{s2}, SLD_{solv}: scattering length densities of the core, first shell, second shell, and solvent, respectively; t_1 and t_2: thickness of the first and second shell, respectively; R_{tot} = radius of the core-shell particle; Φ = hard sphere volume fraction. ‡R_{tot} is not a fit model parameter.

**Samples composition**	**PEG-*g*-PVAc (wt%)**	9.5	19.0	28.5	38.0	47.5	57.0		66.5
	**L-carvone (wt%)**	5.0	5.0	5.0	5.0	5.0	5.0		5.0
	**Water (wt%)**	85.5	76.0	66.5	57.0	47.5	38.0		29.5
**Core-shell form factor + hard-sphere structure factor**	R_c **(Å)**	87 ± 0.6	63 ± 0.8	64 ± 1.3	56 ± 0.8	48 ± 0.7	34 ± 1.0	-	-
	σ	0.31	0.31	0.32	0.30	0.35	0.4	-	-
	SLD_{core} (10^{-6} Å^{-2})	8.9	9.2	9.7	9.7	9.7	9.7	-	-
	t_1 **(Å)**	32 ± 0.7	25 ± 0.6	20 ± 1.2	22 ± 0.9	26 ± 0.9	19 ± 1.7	-	-
	SLD_{s1} (10^{-6} Å^{-2})	10.5	10.5	10.5	10.5	10.5	10.5	-	-
	t_2 **(Å)**	43 ± 1.2	40 ± 0.8	20 ± 1.4	14 ± 0.5	8 ± 0.5	18 ± 0.9	-	-
	SLD_{s2} (10^{-6} Å^{-2})	9.5	9.5	9.5	9.5	9.5	9.5	-	-
	$R_{tot} = R_c + t_1 + t_2$ (Å)‡	162	128	104	92	82	71	-	-
	Φ	0.15	0.24	0.33	0.35	0.37	0.32	-	-
**Teubner-Strey model**	**Correlation length, ξ (Å)**	-	-	-	-	-	-	89	70
	**Repeat distance, *d* (Å)**	-	-	-	-	-	-	160	148
	**Amphiphilicity factor,** f_a	-	-	-	-	-	-	-0.6	-0.5

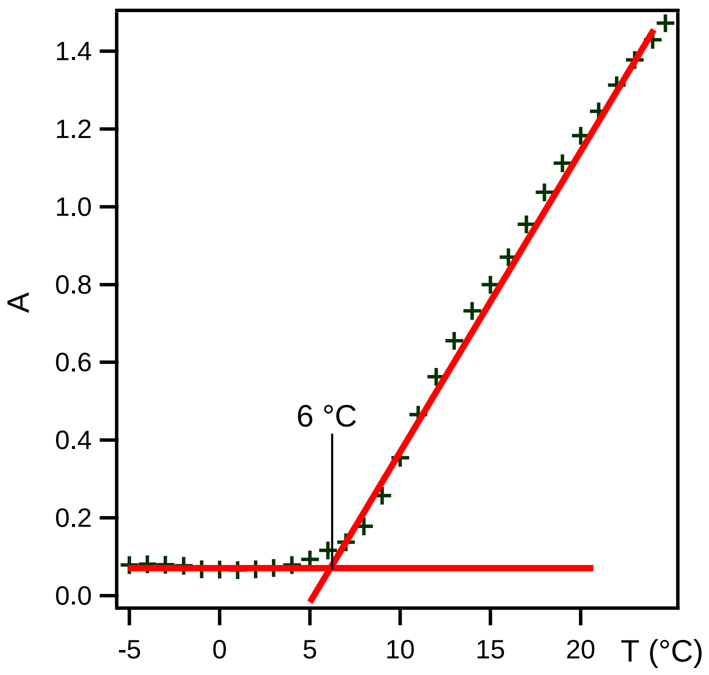

Fig. S5. Plot of the UV-vis absorbance at λ = 500 nm vs. temperature for PEG-*g*-PVAc/PE/water = 9.5/5.0/85.5 %wt. The two slopes were fitted to straight lines; the crossing point corresponds to the cloud point temperature of the polymer in this system.

References

(1) Kinning, D. J.; Thomas, E. L. Hard-Sphere Interactions between Spherical Domains in Diblock Copolymers. *Macromolecules* **1984**, *17* (9), 1712–1718. https://doi.org/10.1021/ma00139a013.

(2) Percus, J. K.; Yevick, G. J. Analysis of Classical Statistical Mechanics by Means of Collective Coordinates. *Phys. Rev.* **1958**, *110* (1), 1–13. https://doi.org/10.1103/PhysRev.110.1.

(3) Teubner, M.; Strey, R. Origin of the Scattering Peak in Microemulsions. *The Journal of Chemical Physics* **1987**, *87* (5), 3195–3200. https://doi.org/10.1063/1.453006.

(4) Koehler, R. D.; Schubert, K. -V.; Strey, R.; Kaler, E. W. The Lifshitz Line in Binary Systems: Structures in Water/C_4E_1 Mixtures. *The Journal of Chemical Physics* **1994**, *101* (12), 10843–10849. https://doi.org/10.1063/1.467833.

(5) Leitão, H.; Telo da Gama, M. M.; Strey, R. Scaling of the Interfacial Tension of Microemulsions: A Landau Theory Approach. *The Journal of Chemical Physics* **1998**, *108* (10), 4189–4198. https://doi.org/10.1063/1.475817.
